# Microglial crosstalk with astrocytes and immune cells in amyotrophic lateral sclerosis

**DOI:** 10.3389/fimmu.2023.1223096

**Published:** 2023-07-26

**Authors:** Matteo Calafatti, Germana Cocozza, Cristina Limatola, Stefano Garofalo

**Affiliations:** ^1^Department of Physiology and Pharmacology, Sapienza University of Rome, Rome, Italy; ^2^Istituto di Ricovero e Cura a Carattere Scientifico (IRCCS) Neuromed, Pozzilli, Italy; ^3^Department of Physiology and Pharmacology, Sapienza University, Laboratory Affiliated to Istituto Pasteur, Rome, Italy

**Keywords:** amytrophic lateral sclerosis (ALS), Astrocyctes, immune cell, inflammation, microglia

## Abstract

In recent years, biomedical research efforts aimed to unravel the mechanisms involved in motor neuron death that occurs in amyotrophic lateral sclerosis (ALS). While the main causes of disease progression were first sought in the motor neurons, more recent studies highlight the gliocentric theory demonstrating the pivotal role of microglia and astrocyte, but also of infiltrating immune cells, in the pathological processes that take place in the central nervous system microenvironment. From this point of view, microglia-astrocytes-lymphocytes crosstalk is fundamental to shape the microenvironment toward a pro-inflammatory one, enhancing neuronal damage. In this review, we dissect the current state-of-the-art knowledge of the microglial dialogue with other cell populations as one of the principal hallmarks of ALS progression. Particularly, we deeply investigate the microglia crosstalk with astrocytes and immune cells reporting *in vitro* and *in vivo* studies related to ALS mouse models and human patients. At last, we highlight the current experimental therapeutic approaches that aim to modulate microglial phenotype to revert the microenvironment, thus counteracting ALS progression.

## 1 Mechanisms of neurodegeneration in ALS

ALS is the most common adult-onset motor neuron disease, affecting the upper and lower motor neurons (MN) in the brain and spinal cord. People with ALS develop muscle weakness and atrophy, leading to paralysis and death from neuromuscular respiratory failure, within 3 to 5 years after onset. Riluzole and the free-radical scavenger edaravone are the only treatments approved to treat ALS patients, which act on survival and rate of progression, respectively.

Traditionally, ALS has been classified as either the sporadic or familial form. Sporadic ALS (sALS) is the most common form, accounting for around 90% of all cases (1–5). Familial, or inherited, ALS (fALS) runs in families and accounts for the remaining 5-10% of cases. Mutations in several genes have been implicated in fALS and contribute to the development of sALS (e.g. superoxide dismutase 1 (SOD1), fused in sarcoma (FUS), TAR DNA binding protein (TARDBP) and chromosome 9 open reading frame 72 (C9orf72) (6–8). Proteins encoded by these genes are involved in several aspects of MN function and in ALS pathogenesis, including protein homeostasis, axonal transport, DNA repair, RNA metabolism, vesicle transport, inflammation, mitochondrial dysfunction, and glial cell function (9–11). In 1994, Gurney et al. developed the first mouse model of ALS, a transgenic model that over-expressed the ALS-associated mutant SOD1^G93A^ and recapitulate some of the key clinical features of human ALS (12). These mice showed evident progressive motor abnormalities and paralysis, microglia acquire an inflammatory phenotype affecting MN death, and myeloid cells expressing mutated SOD1 promote neurotoxicity (8, 13, 14). In another ALS mouse model based on TDP-43 mutations, the TDP-43^A315T^ mice showed pathological aggregates of ubiquitinated proteins in specific neurons and reactive gliosis, with the loss of both upper and lower MNs (15, 16). Abnormal expansion of an intronic hexanucleotide GGGGCC (G4C2) repeat of the C9orf72 gene is ALS’s most frequently reported genetic cause (17–20). Several transgenic mouse models containing the full-length C9orf72 gene show decreased survival, paralysis, muscle denervation, MN loss, and cortical neurodegeneration (21).

Despite the advance of knowledge of the ALS pathogenesis, most of the molecular and cellular mechanisms involved in the progression and development of the disease remain largely unexplored. Recently, a majority of the evidence indicates that ALS is a non-neuronal-autonomous disease (1, 2, 13). Indeed, in this scenario, glia and immune cells build up a complex regulatory network involved in ALS disease, exerting both neurotoxic and neuroprotective effects on neurons. In this review, we summarize the role of non-neuronal cells in ALS pathology, with a particular focus on microglia interplay with astrocytes and peripheral immune cells that orchestrates the ALS microenvironment. We discuss the current therapeutic approaches that aim to modulate microglial crosstalk with non-neuronal cells, and finally look to the future of new therapeutic trials.

## 2 Microglial functions in health and disease

Microglia, which represent ~5–12% of the Central Nervous System (CNS) cells, are the resident macrophages of the CNS (22, 23), that originate from myeloid precursor cells which enter into the CNS during embryogenesis (24), becoming independent self-renewing population (25, 26). The general definition of microglial role describes them as able to perform three essential functions: sensing their environment, maintaining physiological homeostasis, and protecting from self and exogenous stimuli. Microglia are able to adopt a plethora of phenotypes, depending on the surrounding environment, which can differ in the healthy CNS and in various disease states (27–30). Recent *in vivo* imaging studies clearly demonstrate that microglial thin processes continually explore and sample the local environment at steady state (31, 32). Consistently, in the healthy CNS, microglia are necessary for proper brain development, providing trophic support to neurons, removing apoptotic cell debris, regulating neuronal and synaptic plasticity, developmental myelination, and tissue regeneration (33–37). In several pathological conditions, microglia lose their homeostatic molecular signature, resulting in rapid modification of their morphology, transcriptional profile, and phagocytic activity, acquiring a pro-inflammatory profile (38–44); the persistent inflammation can lead to neurotoxicity, and ultimately to neurodegeneration (45–47). Microglia have also been described as active participants in host defense against pathogens and protein aggregates, such as β-Amyloid (Aβ), mutant huntingtin, prions (PrPsc), α-synuclein, oxidized or SOD1 (48–50). In response to CNS insults, microglia initiate a defense program to restore brain homeostasis, through different pattern recognition receptors (PRRs), including toll-like receptors (TLRs), scavenger receptors (SRs), and complement receptor 3 (CR3) (51–58). Furthermore, neurotoxicity dysregulates microglial immunological checkpoints (such as Trem2 and CX3CR1) that normally prevent their overreaction and may help to control inflammation (52). Dysregulation of these pathways increases the risk for Alzheimer’s disease (AD), frontotemporal lobar degeneration (FTLD), frontotemporal dementia (FTD), and ALS (59). Dysregulation of the host-defense pathway further, may also be caused by mutations in specific genes, such as Trem2, HTT, and TDP43, further, resulting in an inflammatory response and neuronal damage (60–62).

### 2.1 Microglia in ALS

Evidence that detrimental microglia contribute to sustaining the inflammation in ALS is observed in imaging studies in patients with ALS, human post-mortem samples, and rodent models of ALS (63–65). Microglia increase the expression of CD14, CD18, SR-A, and CD68 in ALS spinal cord, and CD68^+^ microglial cells are detected in close proximity to MNs (66), and in the brain of ALS patients using Positron emission tomography (PET) imaging (67, 68). Notably, microglia modify their phenotype with disease progression: adult microglia isolated from ALS mouse models at disease onset show a protective/anti-inflammatory phenotype, while microglia isolated from end-stage disease are toxic/pro-inflammatory (69–74). Consistently, in familial ALS (fALS) patients with SOD1 mutations, and in the SOD1-ALS mouse models, microglia affect MN death (75) and promote neurotoxicity (76), as well as regulate the feeding behavior and overall metabolism (77). Microglial-mediated MN death in ALS occurs through an NF-κB-dependent mechanism (75) and by secreting reactive oxygen species and pro-inflammatory cytokines (such as interleukin (IL)-1, IL-6, tumour necrosis factor (TNF)-α) (78, 79). Although a number of studies are in line with the neuroinflammatory and neurotoxic microglial role observed in SOD1 mutated ALS patients and mouse models (8, 13, 14), the functional role of microglia in familial ALS or mouse models with mutated TDP-43 is not clear. In post-mortem human brain tissue from ALS patients and mouse models expressing mutated TDP43, were observed aggregates of ubiquitinated proteins in MNs, astrocytes and microglia, loss of both upper and lower MN and intestinal dysmotility that could induce premature death (15). On the contrary, the suppression of mutated human(h) TDP-43 protein in neurons, dramatically increased the microglial proliferation and changed their morphology and phagocytic activity against neuronal hTDP-43 (15). The partial depletion of microglia using PLX3397, a CSF1R and c-kit inhibitor, failed to recover motor functions in hTDP-43 mice, revealing an important neuroprotective role for microglia (80).

Recent work showed that C9orf72 expression is highest in myeloid cells, and loss of function of the C9ORF72 protein in mice disrupts microglial function and may contribute to neurodegeneration in C9orf72 expansion patients (18, 20, 81–87). Other disease mechanisms that occur in the ALS/FTD with C9orf72 gene mutation include a gain of toxicity mediated through either the RNA itself (88, 89) and/or the translation of aberrant dipeptide repeat (DPR) proteins by a non-canonical translation mechanism called repeat associated non-AUG dependent (RAN) translation (90, 91). Immunoreactive microglia and upregulation of inflammatory pathways have been confirmed in patients with mutated C9orf72 and correlate with rapid disease progression (87). Anyway, since most C9orf72 mouse models do not show ALS motor symptoms, neurodegeneration, or inflammatory response, it is difficult to determine the relationship between C9orf72-specific molecular pathology and ALS.

## 3 Microglial dialogue with non-neuronal cells in ALS

In the CNS, non-neuronal cells play crucial homeostatic functions both in health and diseases. The involvement of these cells in the pathophysiology of ALS is being increasingly characterized. Microglia crosstalk with peripheral immune cells and astrocytes to exert either neuroprotective or adverse effects through a broad range of cell-to-cell interactions.

### 3.1 Microglia – Astrocytes crosstalk

Similar to microglia, during ALS progression, astrocytes adopt neurotoxic properties which actively contribute to disease pathogenesis (92). In fact, both *in vitro* and *in vivo* studies demonstrated that in ALS mouse models, astrocytes with mutated mSOD1 protein exert neurotoxic effects on motor neurons, by releasing pro-inflammatory factors (93). Moreover, reactive astrocytes were described in the post-mortem CNS tissue obtained from ALS patients (94–96). This finding was confirmed further *in vivo* via diagnostic imaging using PET scanning demonstrating cerebral white matter and pontine astrogliosis in ALS patients (97).

In recent years, the crosstalk between astrocytes and other non-neuronal cells has been studied in more detail. The astrocytic transition from neuroprotective to neurotoxic was accompanied by a shift in microglial phenotype, suggesting that astrocytes may be important regulators of microglia activation and neuroinflammation in ALS (98). To date, several studies demonstrated that the detrimental microglia shape astrocyte phenotype in ALS driving disease progression. Both in human and mouse ALS tissues it was found the presence of neurotoxic reactive (A1-like) astrocytes since the early phase of the disease (92, 99–101). Moreover, microglia modify their phenotype as the disease progression: indeed, adult microglia isolated from ALS mouse models at disease onset show a protective/anti-inflammatory phenotype, while microglia isolated from end-stage disease are toxic/pro-inflammatory (67–72).

However, controversial results have been obtained on which cell population, between microglia and astrocytes, acquires a pro-inflammatory/neurotoxic phenotype during ALS progression. Indeed, Alexianu et al. reported that microglia activation precedes astrocyte reactivity (102). On the contrary, another study suggested that astrogliosis is present since the early symptomatic stage, while prominent microgliosis is only evident at the late phase (103).

Astrocytes have been shown to shape the microglial phenotype according to disease stage, modulating neuroprotective and neurotoxic functions in the pre-symptomatic and symptomatic phases of ALS, respectively (98). In the SOD1^G93A^ ALS mouse model, astrocytic NF-κB activation drove microglial proliferation and leukocyte infiltration in the CNS (98). This response was initially beneficial by prolonging the pre-symptomatic phase, but it became detrimental in the symptomatic phase, accelerating disease progression. Specifically, in the pre-symptomatic phase astrocytic NF-κB activation in SOD1 mouse models induced a Wingless-related integration site (Wnt)-dependent anti‐inflammatory microglial response via nuclear factor kappa-B kinase subunit beta (IKK2), resulting in neuroprotective effects on motor neurons which translated into a delay of motor symptoms (98). However, in the symptomatic phase, NF-κB activation in astrocytes promoted pro‐inflammatory microglial responses (via CD68, TGF‐β, TNF‐α) which accelerated disease progression (98). A recent study indicated astrocytic TGF-β1 as a major molecule modulating microglial phenotype toward detrimental one (104). Astrocyte-specific overproduction of TGF-β1 in SOD1^G93A^ mice interfered with the neuroprotective effects of microglia during the pre- and early symptomatic stages and accelerated disease progression in a non-cell-autonomous manner. This interference resulted in reduced production of neurotrophic factors from microglia and a reduced number of CNS infiltrating T cells. Consistently, the expression levels of endogenous TGF-β1 in SOD1^G93A^ mice negatively correlated with overall life expectancy, while the administration of a TGF-β signaling inhibitor extended it (104). These findings raise TGF-β1 to an important determinant of disease progression in ALS.

On the other hand, many pieces of evidence suggest that the activation of microglia precedes the reactivity of astrocytes in ALS (105). Brites and colleagues demonstrated that microglia respond earlier than astrocytes to cell stress or damage by activating NF-κB and mitogen-activated protein kinase (MAPK) signaling pathways, thus leading to the release of pro-inflammatory cytokines (e.g., TNF-α and IL-1β) (105). These cytokines were shown to exert an inhibitory effect on Cx-43 expression, the main constitutive protein of astrocytes’ gap junctions, therefore hampering communication between astrocytes, and possibly interfering with their neuroprotective role (106). In addition, cell–cell contacts and microglial-derived soluble mediators are necessary for astrocytes to fully respond to lipopolysaccharide (LPS) insult and Toll-Like Receptor (TLR) ligation (11413), suggesting that microglia may exert a permissive effect on astrocyte pro-inflammatory activation. Liddelow et al. demonstrated that the microglial derived-pro-inflammatory cytokines IL-1α, TNFα and complement component C1q42 are necessary and sufficient to induce pro-inflammatory astrocytes in mice (92). Consistently, a triple knock-out of these factors in IL-1α^−/−^ TNFα^−/−^C1qa^−/−^ SOD1^G93A^ mice led to a drastic reduction in the number of reactive astrocytes, improving lifespan and delaying motor neuron loss and disease progression (92). This finding further supports the hypothesis of a microglia-to-astrocyte polarization.

Since an intimate interaction/communication between microglia and astrocytes occurs in ALS, a better understanding of their crosstalk could help to define potential therapeutic strategies targeting the glia in ALS.

### 3.2 Microglia – Natural killer cells crosstalk

NK cells contribute to ALS progression by interacting with CNS resident cells and peripheral immune cells. Increased numbers of NK cells have been found in the peripheral blood and CNS of ALS patients (107), and a rich infiltrate of NK cells has been described in the CNS of SOD1^G93A^ mouse models (5). The NK cells - microglia crosstalk has been recently characterized, highlighting the importance of this interaction in the pathogenesis of ALS. Indeed, in NK cell-depleted hSOD1^G93A^ and TDP43^A315T^ microglia acquired a typical neuroprotective morphology, covering a wider parenchymal region and increasing the branches number (108). Moreover, NK cell-depleted hSOD1^G93A^ mice showed a reduction in microgliosis, indicated as the number of microglia in the ventral horns of the spinal cord (108). In the absence of NK cells, microglia reduced the expression of genes associated to a pro-inflammatory phenotype, including IL-6, IL-1β, TNF-α, with the simultaneous increase of expression of the anti-inflammatory (Chil3, Arg-1, and TGF-β), antioxidant (Msod1) and neuroprotective (P2yr12, Trem2, Kcnn4, Bdnf, IL-15) markers (108). The molecular link that drives the crosstalk between microglia and NK cells in ALS is the IFN-γ produced by infiltrated NK cells during the pre-symptomatic stage of disease. Accordingly, the IFN-γ immunodepletion (via IFN-γ-blocking antibody XMG1.2 administration) had consequences similar to NK cell depletion on microglial phenotype, switching them toward an anti-inflammatory phenotype (108). Lastly, this NK cell-mediated modulation of microglia resulted in an increased number of motor neurons in the ventral horn of spinal cord, and affected survival and onset time both in SOD1^G93A^ and TDP43^A315T^ mouse models (108), These results were further validated in an elegant study (109) exploiting Natalizumab, a blocking antibody for the α4 integrin (anti-VLA-4) (110, 111), to reduce the transfer of peripheral immune cells to the CNS of the hSOD1^G93A^ ALS mouse model. In the lumbar spinal cord of Natalizumab-treated mice was found a reduced number of NK cells and, accordingly, microglial cells reduced the expression of pro-inflammatory markers (IL-6, IL-1β and tnf-α), and IFN-γ level was significantly reduced compared to vehicle-treated hSOD1^G93A^ mice (108). However, Natalizumab treatment showed more effects on the modulation of the inflammation in the ALS microenvironment, suggesting a more complex scenario due to the role of different peripheral immune cells infiltrated in the CNS. Overall, these results point toward the importance of microglia-NK cells crosstalk modulation to reduce motor neuron loss in ALS.

### 3.3 Microglia - T lymphocytes crosstalk

Activated T cells are present in the CNS at a steady state to perform immunological surveillance (112) and provide immunological responses that are modulated by cell to cell signaling (113). Infiltration of CD4^+^ and CD8^+^ T lymphocytes has been documented in the brain and spinal cord of ALS patients (114, 115). Specifically, perivascular and intraparenchymal CD4^+^ helper T cells were found to surround degenerating corticospinal tracts, while ventral horns were enriched with both CD4^+^ helper and CD8^+^ cytotoxic T cells. The lymphocytic infiltration did not correlate with the rate of progression or stage of the disease in ALS patients (115); on the contrary, in transgenic mice expressing mutant SOD1^G93A^, the number of CD4^+^ and CD8^+^ T cells infiltrating the spinal cord increased as the disease progressed (116, 117). Multiple levels of evidence suggest that CD4^+^ helper T cells exert neuroprotective functions, especially in the initial phases of the disease process (116, 118), while CD8^+^ cytotoxic T cells present at later phases of the disease are possibly neurotoxic (119, 120). T cell functional profiles are, at least in part, shaped by a complex dialogue with microglia and neurons, as explained below.

#### 3.3.1 Microglia - CD4^+^ T lymphocytes crosstalk

CD4^+^ T cells comprise multiple functionally distinct cell populations that regulate different functions, classified as Th1, Th2, regulatory T cells (Tregs), and Th17 cells (121). Although the role of CD4^+^ T cells in ALS remains controversial, the putatively protective effect of these cells on MNs is widely accepted (122–124). A major insight into the role of CD4^+^ T cells came from Beers & al., who bred immunodeficient mice lacking functional lymphocytes or functional CD4^+^ T cells with mSOD1^G93A^ transgenic mice and performed selective reconstitution experiments with bone marrow transplants (116). The lack of functional CD4^+^ T lymphocytes resulted in a faster disease progression characterized at the molecular level by the upregulated expression of pro-inflammatory functional markers like NOX2 and pro-inflammatory cytokines, while reconstitution of CD4^+^ T lymphocytes prolonged survival and inhibited the acquisition of pro-inflammatory phenotype in microglia (116). The absence of functional CD4^+^ T cells in mSOD1^G93^ mice reduced the mean survival time, supporting the neuroprotective role of these lymphocytes. The fractalkine receptor (CX3CR1), a chemokine receptor expressed by microglia, monocytes, dendritic cells, and subsets of T cells, was involved in microglial neurotoxicity (125), and consistently, was reduced in mice lacking CD4^+^ T cells and increased following bone marrow reconstitution (125).

Within the CD4^+^ T lymphocyte subsets, endogenous Tregs are particularly associated to neuroprotection in ALS, with a time-specific effect (122, 123). Tregs were found to be increased in spinal cords of mSOD1 mice after disease onset, accompanied further by increased expression of IL-4 and higher number of neuroprotective/anti-inflammatory microglia (122). During the progression of the disease, there was a loss of Forkhead box P3 (FoxP3) expression in Tregs, with a concomitant reduction of IL-4 level (122). Passive transfer of Tregs from donor mSOD1^G93A^ mice in the early phase of the disease, sustained IL-4 levels and anti-inflammatory microglia, delaying the onset of symptoms and increasing the survival of recipient mSOD1^G93A^ mice (116, 122).

In ALS patients, neuroinflammation can be attributed to the impaired suppressive function of Tregs in addition to their decreased numbers (123, 126). Indeed, mutated SOD1 Tregs were less effective in suppressing effector T cells (Teff) proliferation (123). With the progress of diagnostic imaging, PET of activated microglia in ALS patients offers a potential opportunity to assess Treg-mediated neuroprotection (63, 67, 127). While Treg and anti-inflammatory microglia increase in the early stage of ALS (128–130), Th1 and pro-inflammatory microglia increased the inflammation in the microenvironment in the later stage of ALS (131, 132). Accordingly, a parallel shift from a neuroprotective Treg/anti-inflammatory response to a neurotoxic Th1/pro-inflammatory response has been postulated during ALS progression by Zhao et al. (132). In the mSOD1 mouse model, a Treg/anti-inflammatory response dominates the initial slowly progressing phase of the disease, as Tregs suppress microglial toxicity and SOD1 T effector cells through IL-4, IL-10 and TGF-β (132). During ALS progresses, the immune response switches to a deleterious Th1/pro-inflammatory response, where the interaction between Th1 and microglia enhances pro-inflammatory responses, including the release of TNF-α, IL-6, and IL-1β, and downregulates Treg suppressive functions (132).

Overall, these data support the concept of a well-orchestrated and complex dialog among microglia and CD4^+^ T cells, suggesting that different CD4^+^ T lymphocyte subsets play different roles in shaping microglial functions during ALS progression.

#### 3.3.2 Microglia – CD8^+^ T lymphocytes crosstalk

In the peripheral blood of ALS patients, cytotoxic CD8^+^ T cells number was found to be significantly increased, suggesting a systemic immune activation (133). However, the role of these cells in the progression of ALS remains difficult to decipher (134).

Particularly, microglia-CD8^+^ T cell crosstalk is fundamental to drive the inflammation in ALS affected regions (120). Specifically, major histocompatibility complex I (MHCI) depletion in resident microglia or the lack of CD8^+^ T cell infiltration in the spinal cord of β2 microglobulin-deficient hSOD1^G93A^ mice (which express little if any MHCI on the cell surface and are defective for CD8+ T cells) delayed motor symptoms and prolonged the survival mean time, suggesting that microglia interact with infiltrated CD8^+^ T cells through MHC complex, promoting MN death in ALS. Moreover, the level of CD68^+^ microglia was lower in the spinal cord of β2 microglobulin-deficient hSOD1^G93A^ mice suggesting that the MN preservation is due to a lack of interaction with CD8^+^ T cells (120). Interestingly, β2 microglobulin-deficiency in the peripheral nervous system (i.e. sciatic nerve) impaired motor axon stability and anticipated the onset of muscle atrophy, delineating regional differences in the role of MHCI and CD8^+^ T cells in the pathogenesis of ALS (120).

### 3.4 Microglia – Monocytes/macrophages crosstalk

In ALS patients, peripheral monocytes infiltrate the CNS (66), and the monocytes isolated from peripheral blood of ALS patients show a pro-inflammatory profile (135). Furthermore, the degree of systemic monocyte/macrophage activation directly correlates to the rate of disease progression (136). In the hSOD1^G93A^ mouse model, inflammatory monocytes infiltrate the ALS affected regions (137), and their progressive recruitment to the spinal cord correlates with neuronal loss (137). Prior to disease onset, monocytes expressed a polarized macrophage pro-inflammatory phenotype (M1 signature), which included increased levels of chemokine receptor CCR2. This receptor normally interacts with the ligand CCL2, controlling the migration and infiltration of CCR2-expressing monocytes/macrophages in a process implicated in multiple neurodegenerative diseases. Butovsky et al. demonstrated that CCL2 expression by microglia increases as ALS progresses (138). Mouse monocytes fall into two phenotypically distinct subsets: Ly-6C^hi^ (which are CCR2^+^) and Ly-6C^lo^ (which are CCR2^−^), corresponding to human CD14^hi^CD16^−^ and CD14^+^CD16^+^ monocytes, respectively (138). Ly6C is a GPI-linked protein of the Ly6 family, which is found mostly in inflammatory monocytes (139). Accordingly, hSOD1^G93A^ mouse treatment with anti-Ly6C monoclonal antibody reduced the number of monocytes recruited to the spinal cord, diminished neuronal loss, and extended survival (137).

Recently, a study by Chiot et al. (140) investigated the crosstalk between peripheral macrophages and microglia in ALS. Targeted gene modulation of the reactive oxygen species pathway in peripheral myeloid cells of hSOD1^G93A^ mice, reduced both peripheral macrophage and microglial activation, and delayed the onset of motor symptoms (140). Specifically, the chemotherapy agent busulfan was used to induce myeloablation, followed by bone marrow transplantation in which mutant SOD1-expressing macrophages were replaced with macrophages genetically modified with less neurotoxic properties (via downregulating of Nox2 or overexpression of wild-type Sod1). In this model, resident microglial cells acquired an anti-inflammatory/protective phenotype and a reduction was found in microgliosis in the spinal cord (140). These results indicate that the modification of infiltrating monocytes/macrophages suppresses neurotoxic microglial responses in ALS, suggesting direct or indirect crosstalk between these two cell populations. The mechanisms underlying this crosstalk are not yet clear, but the authors suggested that replacing of inflammatory peripheral monocytes/macrophages could pave the way for a new therapeutic approach for ALS patients.

## 4 Currently approved therapies in ALS

The complex pathogenesis in ALS, coupled with its clinical and molecular heterogeneity, resulted in too many failed attempts at drug discovery and development. The foundation for failure includes the wrong target, route of administration, outcome measures, and the many different pathogenic mechanisms at play in different patients (141). Drugs undergoing clinical trials are available on the ALS Association website (https://www.neals.org/als-trials/search-for-a-trial/). To date, there are two FDA-approved drugs for ALS: riluzole and edaravone. Both drugs have a relatively small efficacy in delaying motor function deterioration, and their effectiveness is limited during early stages of the disease (142).

Riluzole was the first FDA drug approved for clinical use in 1995. This drug blocks glutamate release and therefore glutamatergic neurotransmission in the CNS, exerting neuroprotective function as it dampens pathological excitotoxicity in ALS (143). Additional proposed mechanisms of action include an indirect antagonism of glutamate receptors in addition to the inactivation of neuronal voltage-gated Na^+^ channel (144). Edaravone was the second FDA approved ALS-specific drug, in 2017. Edaravone is a neuroprotective drug with broad free radical scavenging activity that protect neurons, glia, and vascular endothelial cells against oxidative stress (145).

### 4.1 Therapies targeting neuroinflammation and microglial crosstalk with peripheral immune cells

Multiple compounds with immune-modulatory properties have been reported to affect the crosstalk between microglia and immune cells. Although promising in the mouse models of ALS, preclinical results have so far failed to translate into meaningful clinical outcomes (146, 147). Most efforts in the development and application of immune-modulatory drugs in ALS aimed at reducing pro-inflammatory and neurotoxic immune responses. Among the therapies recently developed to target neuroinflammation and microglia phenotype in ALS, the following demonstrated significant benefits in preclinical studies and have already or are soon to be translated to clinical trials (**Table 1**).

**Table 1:** Therapies targeting neuroinflammation and microglial crosstalk with peripheral immune cells in ALS.

Drug	Mechanism	Target cells	Trial number	Phase
dl-3-*n*-Butylphthalide (NBP)	Modulation of mitochondrial oxidative stress, apoptosis and autophagy	Microglia, Motor Neurons	ChiCTR-IPR-15007365	II
Cannabinoids	Anti-glutamatergic, antioxidant and anti-inflammatory actions	Microglia	N/A	N/A
Ibudilast (MN-166)	Anti-inflammatory and neurotrophic actions	Microglia	NCT02238626 NCT04057898	II IIb/III
Masitinib	Anti-inflammatory; modulation of aberrant microgliosis	Microglia	NCT02588677 NCT03127267	II/III III
Minocycline	Anti-inflammatory	Microglia, Motor Neurons	NCT00047723	III
NP001	Anti-inflammatory	Microglia	NCT01091142 NCT01281631 NCT02794857	I II II

N/A, not applicable.

- dl-3-*n*-Butylphthalide (NBP) is a small molecule compound showing neuroprotective effects via multiple mechanisms, including modulation of mitochondrial oxidative stress, apoptosis and autophagy (148). In hSOD1^G93A^ mice, treatment with NBP extended survival by attenuating microglial activation and motor neuron loss (149, 150). A randomized trial (Chictr.org.cn Identifier: ChiCTR-IPR-15007365) of NBP in the treatment of ALS patients was conducted in China. The preliminary results indicated that NBP did not improve the ALS Functional Rating Scale (ALSFRS)-R score in patients with ALS (151).
- Cannabinoids exert anti-glutamatergic, anti-oxidant and anti-inflammatory actions through activation of the CB (1) and CB(2) receptors, whereby receptor activation reduces pro-inflammatory microglia, decreasing the microglial secretion of neurotoxic mediators (152, 153). In hSOD1^G93A^ mice, treatment with WIN-55,212-2, a cannabinoid agonist with higher affinity to the CB2 than the CB1 receptor (154), and the Selective CB2 receptor agonist AM-1241 significantly delayed disease progression and increased mean survival time (155, 156). Although these promising results, a meta-analysis of the studies conducted on murine models concluded that animal studies have moderate to high risk of bias and are highly heterogeneous. Therefore, more standardized studies on cannabinoids are necessary before bringing these compounds to the clinic (157).
- Ibudilast (MN-166) is a non-selective phosphodiesterase 4 inhibitor with a neuroprotective effect primarily mediated by the inhibition of inflammatory mediators and the upregulation of neurotrophic factors in pro-inflammatory microglia (158). Two clinical trials with ibudilast have been completed in ALS patients, and one is currently ongoing. The first Phase II trial (ClinicalTrials.gov Identifier: NCT02238626) evaluated the safety, tolerability and clinical responsiveness of ibudilast co-administered with riluzole. The study showed good safety and tolerability but no overall difference in disease progression between ibudilast and placebo treatment arms. Subgroup analysis suggested that patients with bulbar or upper limb onset might have more benefit from the compound (159). A Phase IIb/III study, the COMBAT-ALS study is currently recruiting on North America in order to evaluate the pharmacokinetics, safety and tolerability and assess the efficacy of ibudilast on function, muscle strength, quality of life and survival in ALS (ClinicalTrials.gov Identifier: NCT04057898).
- Masitinib is a tyrosine-kinase inhibitor whose oral administration was shown to control aberrant microgliosis, abrogate neuroinflammation and slow disease progression in the hSOD1^G93A^ mice (160). The primary analysis of a randomized Phase II/III trial testing masitinib in combination with riluzole for the treatment of ALS patients (ClinicalTrials.gov Identifier: NCT02588677) showed a significantly slowed functional decline, although there was no discernible difference in overall survival between the two arms (161). Long-term survival analysis indicated that oral masitinib prolonged survival by over 2 years as compared with placebo, provided that treatment started prior to severe impairment of functionality (162). A subsequent phase III clinical trial is currently ongoing (ClinicalTrials.gov Identifier: NCT03127267).
- Minocycline is a second-generation tetracycline antibiotic, capable to penetrate the blood-brain barrier, with anti-inflammatory effects independent of its antimicrobial activity. The compound has been demonstrated to dampen microglial activation (163) and apoptosis by inhibiting mitochondrial permeability-transition-mediated cytochrome c release (164). The compound delayed disease onset and extended survival in the hSOD1^G93A^ and hSOD1^G37R^ transgenic models of ALS (164–166). However, a subsequent randomized placebo-controlled phase III trial (ClinicalTrials.gov Identifier: NCT00047723) disproved any efficacy of the compound in patients, reporting an ALSFRS-R score deterioration faster in the minocycline group than in the placebo group, along with a higher incidence of adverse events (167).
- NP001 is a highly purified form of sodium chlorite, targeting inflammatory macrophages by down-regulating the Nuclear Factor kB (NF-kB) inflammatory pathways (168). Preliminary studies in hSOD1^G93A^ mice showed a significant increase in life expectancy compared to control (169). A phase I trial in ALS patients (ClinicalTrials.gov Identifier: NCT01091142) showed that NP001 was generally safe and well tolerated, and caused a dose-dependent reduction in expression of the pro-inflammatory marker CD16 (170). Two subsequent randomized phase II trials (ClinicalTrials.gov Identifier NCT01281631, NCT02794857) suggested that NP001 slowed the progression of ALS symptoms in a subset of patients with marked neuroinflammation (171). Combined *post hoc* analysis did not show significant differences between placebo and active treatment but identified a 40‐ to 65‐y‐old subset in which NP001‐treated patients demonstrated slower declines in ALSFRS‐R score compared with placebo (172).

## 5 Conclusions

**Figure 1:**
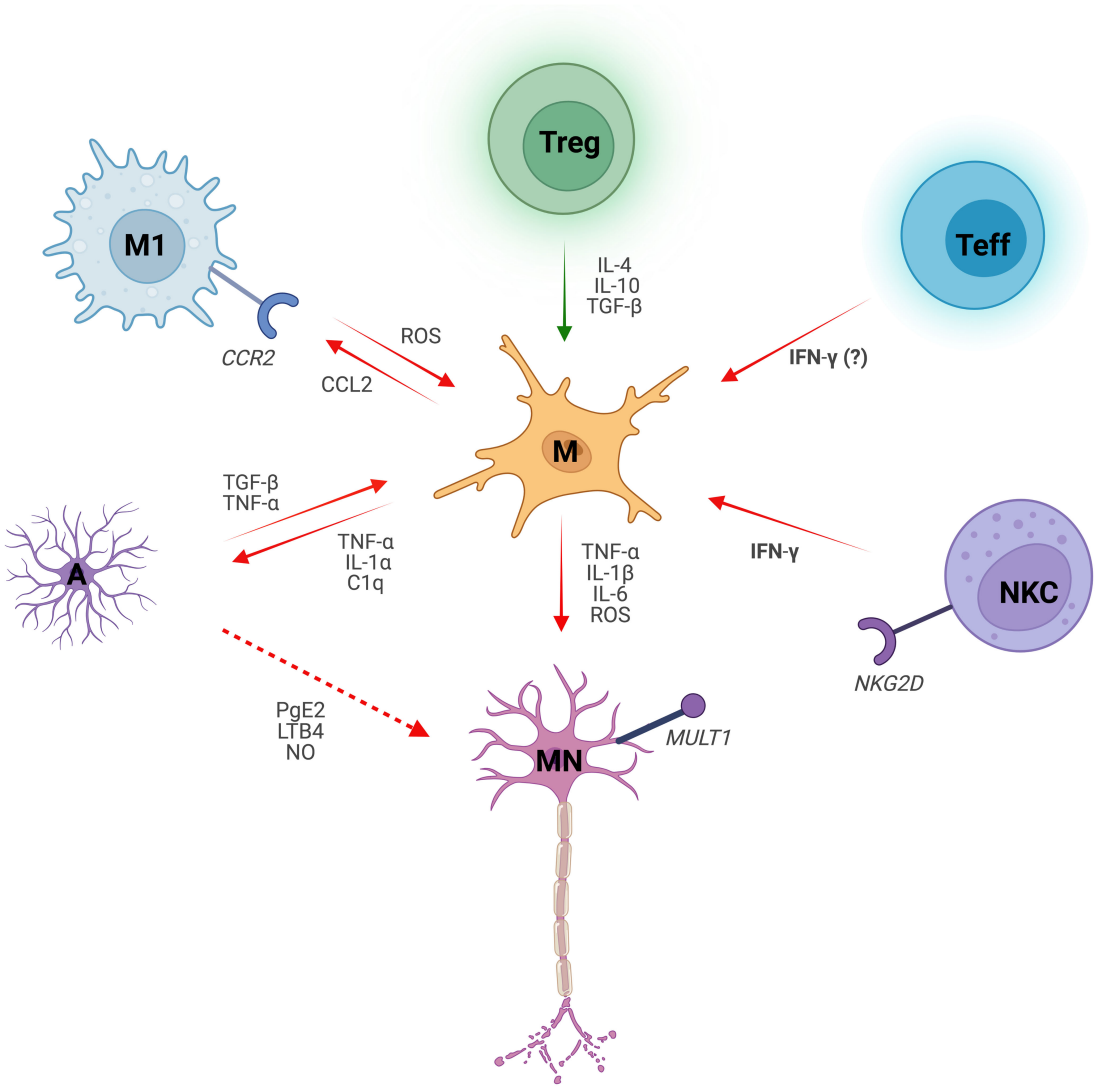
*Microglial dialogue with non-neuronal cells in Amyotrophic Lateral Sclerosis.* Microglia (M) induce motor neuron (MN) degeneration in ALS by secreting reactive oxygen species (ROS) and pro-inflammatory cytokines, such as Interleukin 1 beta (IL-1β), Interleukin 6 (IL-6) and Tumor Necrosis Factor (TNF-α). Microglial crosstalk with non-neuronal cells shapes their phenotype, either skewing it towards a pro-inflammatory (red arrows) on anti-inflammatory (green arrows) phenotype. Microglial-derived pro-inflammatory cytokines Interleukin 1 alpha (IL-1α), TNFα and complement component C1q induce pro-inflammatory astrocytes (A). Conversely, activated astrocytes promote inflammatory microglial responses via Transforming Growth Factor β (TGF-β) and TNF‐α. Reactive astrocytes also exert toxic effects on MNs by secreting inflammatory mediators such as Prostaglandin E2 (PgE2), Leukotriene B4 (LBT4) and nitric oxide (NO). Chemokine ligand 2 receptor (CCR2)-expressing macrophages (M1) are recruited by the Chemokine Ligand 2 (CCL2) released by microglia. ROS pathway in classically activated macrophages induces microglial activation. Regulatory T cells (Treg) suppress microglial toxicity as well as other immune cells (not shown) through Interleukin 4 (IL-4), Interleukin 10 (IL-10) and TGF-β. Notably, TGF-β effect on microglia is context- and cell-dependent. Microglia-CD8+ Effector T cell (Teff) crosstalk drives neuroinflammation in ALS, with Interferon gamma (IFN-γ) secreted by the latter likely playing a role. Infiltrated Natural Killer Cells (NKC) instruct microglia towards an inflammatory profile by the release of IFN-γ. Additionally, NKCs are neurotoxic to MNs via NKG2D - NKG2D ligand (MULT1) interaction.

The crosstalk between immune cells and glia contribute to MN degeneration in ALS. Despite the advance in the scientific findings aimed to unravel the molecular and cellular mechanisms that induce MN to death, ALS persists without effective therapy that improves motor symptoms and increases the life of patients. In ALS microglia promote a pro-inflammatory microenvironment, supported by neurotoxic astrocytes and infiltrated lymphocytes and macrophages that exert an effective immune reaction against MNs (**Figure 1**) (92, 96–108, 120, 140, 173).

Here we review the state-of-art regarding this fascinating cellular communication, highlighting the current hypothesis that modulating the interaction of microglia with astrocytes and immune cells could represent a promising therapy. It is crucial to keep improving the biological knowledge of ALS and the interplay with resident and infiltrating immune cells in order to understand the cell-to-cell communication mechanisms and their role in driving disease pathogenesis. At last, we discuss the current experimental approaches that aim to modulate microglial phenotype to modulate the inflammation in the CNS counteracting ALS progression.

The possibility to integrate these exciting discoveries with new combination therapies will open new tools to treat this devastating disease.

## Author contributions

MC and GC wrote review. CL and SG supervised, wrote and edited the review. All authors contributed to the article and approved the submitted version.
